# Rethinking communications for governance of malaria programs

**DOI:** 10.1371/journal.pgph.0001132

**Published:** 2023-07-31

**Authors:** Jimmy Opigo, Anya L. Guyer

**Affiliations:** 1 National Malaria Control Division, Ministry of Health, Kampala, Republic of Uganda; 2 Consultant, Boston, Massachusetts, United States of America; PLOS: Public Library of Science, UNITED STATES

## Abstract

Malaria has, to a great extent, become normalized and accepted as inevitable. To resume global progress on malaria elimination, national malaria programs in many malaria-endemic countries urgently need to add new tactics. The global COVID-19 experience has demonstrated that it is possible to rapidly shift health programming and governance. In this essay we argue that a key to transforming malaria programming is stronger and more strategic communications to bring malaria to the forefront. Our concept of communications goes beyond the typical malaria behavior change communication or information, education and communication campaigns; to truly have an impact on improving the malaria situation in the countries where it is most entrenched, malaria program staff and advocates must also focus more on strategic communications to rally the full range of stakeholders to prioritize malaria. We searched two databases of peer-reviewed literature and one malaria-focused journal for examinations of strategic communications for malaria governance and found no publications that deal directly with the topic. This paper therefore proposes a framework for strategic communications for malaria governance that involves five key elements: knowing the audience, defining the message, designing a medium, identifying a messenger, and selecting the timing. Throughout the essay, we draw on experiences from Uganda, where one of the authors leads the country’s National Malaria Control Division. Strategic communications can trigger improvements in malaria control by driving and supporting decision-making by individuals and leaders. Further, strategic communications is a tool used to improve policy, mobilize resources, and serve as the management glue that holds a malaria program and team together as they move their nations towards malaria elimination.

## Introduction

Malaria is one of humankind’s oldest and most persistent health challenges. As a result of its longevity, the morbidity and mortality malaria causes have, to a great extent, become normalized and accepted as inevitable in many countries, particularly in sub-Saharan Africa. Preventing malaria is widely treated as a voluntary matter of individual responsibility—through the use of bed nets or removing breeding sites from around homes; once these measures become routine they are often not pursued aggressively,. Among health care providers in endemic countries, treating malaria is considered “business as usual” rather than a crisis requiring urgent and society-wide interventions. Research into new approaches and tools for vector control and disease treatment is initiated and driven by academic questions and industry priorities, rather than by frontline malaria programs. In all, for most people living in malaria-endemic countries, as well as for most global health professionals, malaria is neither a new and interesting topic nor a particularly exciting or engaging challenge.

Yet as malaria experts and many others in global health are well aware, malaria should be a major and urgent cause of concern for governments, health care providers, global health policy advocates and local communities around the world. In this paper, we argue that in order to once again bring malaria to the forefront of health improvement and sustainable development efforts in endemic countries requires going beyond technically sound interventions or increasing funding levels. It also requires going beyond efforts to inform and educate people at risk of malaria. To truly have an impact on improving the malaria situation in the countries where it remains entrenched, malaria program staff and advocates must also utilize strategic communications in order to rally the full range of stakeholders to prioritize working effectively toward eliminating malaria. Effective communications can trigger improvements in malaria control by driving and supporting decision-making by individuals and leaders. It is a tool used to improve policy and mobilize resources. And it serves as the management glue that holds the malaria program and team together and supports them to maintain strategic interests, coherence and focus.

This concept of “strategic communications” related to malaria goes beyond how the term is traditionally used in malaria programming. Typically, malaria programs refer to communications as a program activity—namely through behavior change communication (BCC) or information, education and communication (IEC) campaigns designed to inform people in communities about malaria prevention and treatment options. We focus instead on communication as a “soft skill” that leaders can develop to use as a tool to make national malaria programs more effective in advocating for and carrying out their work. Throughout this essay, we draw on experiences from Uganda, where one of the authors leads the country’s National Malaria Control Division.

Ugandans experience 5% of all global malaria cases and 3% of all malaria deaths, despite accounting for less than 0.05% of the global population [1]. Estimates vary, but malaria infections are the cause of approximately 40% of outpatient visits to clinics around the country and up to 20% of hospitalizations [2, 3]. In a recent study conducted in five clinics in five high malaria-burden districts, malaria was suspected in over 73% of all outpatient visits and nearly 70% of those tested were positive for the disease. These proportions were slightly higher among children under the age of five years [4]. The deaths of more than 10,000 people in Uganda were attributable to severe malaria in 2019 [1]. The problems malaria causes persist despite notable progress made in previous years in controlling the disease, the existence of significant scientific, biomedical and traditional knowledge about preventing malaria by preventing mosquitoes from breeding near and biting humans, and the existence of effective treatments. Uganda’s experience shows that complex efforts to control, eliminate, and ultimately eradicate malaria must be long-term endeavors, not short-term interventions.

Why does malaria remain entrenched in Uganda, as in so many places, despite years of malaria control programming? Why have national malaria interventions not received sufficient and sustained attention and support from political and community leaders? Why are many national malaria programs understaffed and under-resourced, even when funding is available to countries from the Global Fund to Fight AIDS, Tuberculosis and Malaria and other bilateral and private donors? And why has global progress in reducing the burden of malaria slowed in recent years? We argue that these gaps represent failures in local, national, and global governance of malaria interventions. Malaria governance comprises the governmental and institutional structures and processes that: identify malaria as a priority; set ambitious but feasible goals for reducing the burden; and, equip and hold people and institutions accountable for accomplishing the defined objectives. The governance challenges that impede further progress on malaria elimination arise from many factors rooted in history, science, advocacy and culture.

We further argue that a key element of improving malaria governance is improving the strategic communications capacity of the staff of malaria programs. When we conducted a literature review on the topic, as described below, we found no examinations of such efforts. Thus in the rest of this paper we propose a framework for studying this topic further and present our case for prioritizing strategic communications for malaria governance. Among other shifts, malaria programs have to undertake stronger and more strategic communications to promote policies that would support meaningful progress towards the vision of eliminating malaria. The global COVID-19 experience has demonstrated these kinds of shifts are possible; once key stakeholders prioritize dealing with a disease and its societal consequences, the development of technology, provision of funding, deployment of human and other resources, and accountability structures all swiftly follow.

## A gap in the literature

We conducted searches in the PubMed and Web of Science databases for peer-reviewed literature on strategic communications for improving malaria governance using various combinations of the terms “malaria”, “communication”, “policy”, “governance”, and “advocacy.” The vast majority of the articles we found focused on using BCC or IEC strategies with beneficiary communities, and the few we reviewed in detail proved to be only peripherally relevant. We also did a targeted search for the key terms in *Malaria Journal*. Again, the vast majority of the publications focused on BCC or IEC interventions. The remaining publications focused on advocating for policy changes in light of emerging research and global-level policy architecture.

We concluded that almost nothing has been published in the scientific literature about the use of strategic communications by malaria programs to engage stakeholders and foster long-term support and political commitment to the fight against malaria. This gap in the literature reflects our empirical observations about the priorities of malaria research and control programs in many endemic countries: they focus on using their resources on interventions, rather than on more nebulous concerns such as management and communications. However, we believe that in order to resume making progress towards malaria elimination, the malaria community must examine these issues. In the rest of this paper, we present a set of concepts and terms related to communications for malaria governance that we hope can become the subject of both additional research and capacity development among national malaria programs. We propose that utilizing and elaborating on this framework for strategic communications for malaria governance would enable national malaria programs to reach new levels of effectiveness.

## Strategic communications for malaria governance

Throughout this essay, the term “strategic communication” goes beyond IEC, BCC, and means of communications to encompass the full range of both formal and informal activities and mediums by which human beings convey and receive information and messages. In our usage, strategic communications is the use of communication to promote an agenda—such as eliminating malaria morbidity and mortality—by engaging with current and potential stakeholders. Strategic communications is key to building a collective sense of purpose and urgency in a complex and dynamic world.

In Uganda, national malaria control strategies are created by technical experts at the Ministry of Health, often with technical support from international malaria experts. In recent years, these were codified in The Uganda Malaria Reduction Strategic Plan 2014–2020 [5]. A review completed in 2017 identified many shortcomings in the national malaria program and recommended a complete program reorientation to make it more focused, holistic and multi-sectoral, and therefore more effective.

Outlining good technical strategies, however, was only the first step. The program’s leadership realized that in order to make change, they had to move beyond asking only, “How should the national malaria control program achieve its objective: reducing malaria?” They also had to develop a more complex understanding of malaria governance. Getting the resources to implement the proposed new strategies required convincing national decision makers, who allocate the government’s budget, and the international donors that provide financial support. It also entailed convincing busy health care providers to adapt their practices, persuading stakeholders from other public sector agencies and the private sector to lend their support, encouraging political and traditional leaders to participate, and most of all, engaging with local communities—the intended beneficiaries of the strategies—to gain their trust and understand how malaria fit in with their other priorities. Communications also plays a role in building a strong team to implement malaria programming.

## Components of communications for malaria governance

Constant and purposeful strategic communications is not a program that can be easily encapsulated or quantified. It requires soft skills, including tactical planning and leveraging network opportunities, that are not routinely taught in public health and civil administration training programs. However, it is an essential area for malaria program leadership. Broadly, the key elements of communications are:

- Audience: who needs to receive a given message
- Message: what the program wants the audience(s) and stakeholders to learn, understand, or do
- Medium: how the program delivers these messages to different audiences
- Messenger: who delivers the messages to targeted audiences
- Timing: when the audience is open to receiving messages

Regularly developing clarity about each of these is an important practice. For example, the message of this paper is that in order for malaria programs to be more effective in achieving their goals, they need to build their capacity to engage in strategic communications with a wide range of stakeholders. We want this message to be understood by malaria program managers and their supporters. Our medium is this essay. The messenger is the leader of Uganda’s malaria control efforts, whose experiences in his work have led him to understand this message. And by publishing this piece in conjunction with a global effort on “Rethinking Malaria in the Context of COVID–19,” we hope to reach an audience of people excited to consider new approaches to malaria control and elimination programming. So, how does a national malaria control program engage with the full range of stakeholders to “sell” them on supporting the program to pursue its preferred strategies and achieve its stated goals?

### Component #1: The (segmented) audience

Communications seeks to engage all relevant stakeholders to align their understanding and to convince them to participate in creating a functional partnership with unity of purpose. Stakeholders at all levels, from the local community up to the President’s Office and global donors, need to agree on what the malaria program seeks to do, and how it aims to do it. Then they can coordinate individual efforts in Support of shared goals.

The process begins with defining who the key stakeholders are. Stakeholders can then be “segmented” into “audiences.” Depending on their viewpoints and positions, different audiences will respond to different types of messages, different formats, and different messengers. Tailoring communications requires understanding your audiences, and understanding requires listening. In this context, listening can be interpreted literally, as in meeting with stakeholders to solicit their views and learn about their interests and needs. It can also be understood figuratively: the malaria program can “listen” to stakeholders by assessing their actions and behaviors indicate that they have heard the message and are responding to communications in the way the program intended and desired.

Thus strategic communication flows in both directions. In order to understand the aims, interests, and preferences of any audience, malaria program leaders must also make themselves available and open to listening to their concerns. Open dialogues with audiences have many benefits: most of all, it enables leaders to understand their stakeholders’ opinions and situations, which they can then consider in developing and implementing programs. In addition, two-way communication allows leaders to build interpersonal relationships, earn the respect and trust of audiences, and develop a good reputation. This helps to position the leader and the malaria program overall as a source worth heeding.

#### External audiences

In Uganda, external audiences (outside the malaria program) for governance-related communications from the malaria program go far beyond the Ministry of Health or local health authorities. It also includes:

- **Politicians**, including the President and Parliamentarians. Their support for and engagement in malaria programming signals that the government considers malaria an important issue.
- **National and global policy makers**, such as officials in the Ministry of Health, technical advisors to political leaders, and global experts. They can operationalize priority-setting and resource allocation to the malaria program.
- **Funders and health development/implementing partners**, including the Ministry of Finance, donor agencies, and non-governmental organizations. Their understanding of the malaria program facilitates access to resources and support for financial management.
- **Leaders in other sectors** that are affected by or have a stake in malaria. This may include the private sector, such as industries that rely on healthy workers (especially agriculture and mining), and industries that provide commodities used in malaria prevention and treatment, such as pharmaceutical and vector control companies. It also includes other relevant government sector agencies, including finance, economic development and planning, environment, housing, education, tourism and others.
- **Local-level leaders**, such as District Health and Medical Officers, local authorities, and traditional leaders, who allocate resources locally and have significant influence with their constituents.
- Intended beneficiaries of the program. This audience includes on-the-ground implementers, such as health care providers, community health workers, as well as community members and the general public.

Most of these people and organizations are not only outside of the malaria program, but are also outside of the formal health sector. However, their support is essential for the program to implement its activities. Their support may be political, financial or engagement; in all cases, the program needs to convince stakeholders to make some change in order to successfully reduce malaria. In some cases, communications with external stakeholders are recognized responsibilities of a program manager, such as engaging with advisory boards or donors. However, there are many other (less well-defined but equally important) aspects of external communications that also influence the governance of a malaria program.

#### Internal audiences

Malaria program managers must also be strategic as they engage in internal communications with program staff. Strategic internal communications enable a leader to build a cohesive and motivated team that is informed and aligned with a common mission and goals. Creating internal cohesion requires constant communications for team-building, conflict prevention and management, aligning staff with the organization’s stated priorities, and generally organizing the group to work towards the same goals. Internal communications include participating in group meetings where staff can share their opinions, seeking out one-on-one conversations, displaying the mission and vision of the program widely to reinforce it, and others.

Developing cohesion within the organization is critical so that all members of the group can coordinate and reinforce common messages, activities, and responses to the inevitable changes in the environment where the program is working. Maintaining open internal communications is key in change management and conflict prevention among the team. Strategic communications from leadership emphasizes, fosters and supports all staff members to collect and utilize data and evidence, adopt and adapt innovations, and generally participate in the functions of a responsive, adaptive, and learning organization.

As noted, communications has four other elements: the message, the medium, the messenger, and the timing. To truly communicate with each stakeholder, all of these components must be gotten right: an acceptable messenger must deliver a relevant and comprehensible message in an accessible way at a time when the audience is open to receiving it. The malaria program can develop rough indicators to evaluate whether its governance communications have been received, such as the amount of resources donors commit or the backing provided by key leaders in difficult negotiations.

### Component #2: The message

“If malaria is such a big problem, why doesn’t the health sector truly care about it? After all, we never run out of children’s vaccines, but malaria drugs are often unavailable at the clinics.”

At the most basic level, the key message regarding malaria must be that malaria is conquerable and an urgent priority for the current generation. This message is as essential in improving governance of malaria as it is in promoting use of bed nets or encouraging testing in case of a fever. More explicitly, the national malaria program’s message is: malaria is a big problem, but if government, civil society and individuals alike pay more attention and put more resources into addressing it, it could be tackled effectively. This is urgent: interventions exist for preventing and treating malaria, but we are not deploying them widely, strategically, or intensively enough—and doing so is critical before these options lose their potency. Successful malaria prevention is incumbent on individuals and households taking responsibility and actions to prevent mosquito reproduction, protect people from mosquito bites, and seek care promptly in case of fevers. Developing new approaches to malaria prevention is also critical to transcend the status quo and move towards elimination. These efforts require more investment and innovation from all sectors.

Doing strategic communications requires understanding which parts of the message will resonate with which audiences, and tailoring it to elicit support and align with their own agendas. For example, donors do not like to “throw good money after bad.” That is, they prefer to invest resources in programs that are likely to have positive outcomes or at least are innovative. This creates a problem for malaria programs, as the basic interventions are well known, even if they have not been properly implemented. “Rebranding” the program periodically, highlighting innovations and new determination, can help. In Uganda, the national program has rebranded its work with a new logo and slogans that emphasize action, such as “chase malaria to zero,” “under the net,” Mass Action Against Malaria, Malaria Free Uganda and others.

Messages should be designed to respond directly to the concerns and interests of stakeholders. For example, policy makers and politicians rarely make decisions based exclusively on scientific evidence. They may want to know: Who are the intended beneficiaries of a proposed program? Will a new policy solve human rights, gender, or equity issues? Will malaria programming help lift people out of poverty? Will a policy target disadvantaged populations or the whole country? Will a program have a visible impact during their term of office? The malaria program has to understand these concerns and address them directly in its communications with policy makers and politicians. It is also critically important to report back on the results the malaria program achieves, in order to strengthen stakeholders understanding of how the program operates and how it deploys the resources stakeholders invest.

A final note on the language used in messaging: most malaria programs are staffed by technical officers accustomed to using scientific terms, program jargon and many, many abbreviations such as IRS (Indoor Residual Spraying), ITNs (Insecticide-treated Nets), ACT (Artemisinin-based Combination Therapy) or MDA (Mass Drug Administration). These are generally only understandable to other experts. Instead, malaria programs must learn to use widely accessible words, terms and concepts in order to engage and convince key stakeholders.

### Component #3: The messenger

“We have to fight to catch the attention of the President. Whenever there is a vaccination campaign he drops in and then everybody knows that immunizations are important for child survival. We need to do the same for malaria.”

The national malaria program manager is the key figure when it comes to communications for governance. He or she represents the program to external stakeholders and must build team spirit and boost morale within the program. In both roles, skill in informal communication is often overlooked as a job requirement, but it is critically important. The tone, content and frequency of emails, text messages, and phone calls are important. Participating in networking opportunities such as other organizations’ meetings and events, sending seasonal greetings, meeting for conversation over coffee—these activities may seem time-consuming or even trivial compared with scientific research or bed net campaigns. However, informal contacts convey verbal and nonverbal communication that help create openness on the part of the stakeholders. Their openness ultimately serves the malaria program if their trust and engagement make stakeholders open to receiving the malaria program’s messages.

For some audiences, in fact, the messenger may be the most important factor in whether they accept the message. This may be especially true when dealing with representatives of other sectors (who may not immediately see the relevance of malaria to their endeavors) and with political leaders (who are constantly juggling a multitude of stakeholders who want their attention). In addition to *being* knowledgeable, credible, and ethical, the program staff must also be *perceived* as all of these characteristics. Malaria program managers and other staff must work to establish themselves as reliable sources of information and ideas with positive personas.

In some cases, the program staff should consider identifying other appropriate and acceptable messengers to reach key audiences and convey specific messages. These champions may include influential persons, religious, political and traditional leaders, and celebrities.

In Uganda, until recently, the malaria program within the Ministry of Health was understaffed, with only five people. Comparing this team with larger Ministry teams, such as the one leading HIV and AIDS programming, sent a clear nonverbal message about the lack of priority placed on malaria. While various malaria-specific stakeholders had noted the problem over the years, they had never successfully built up the team. When the current manager came on board in 2016, he saw that building up his team was a necessity, as without more staff, the program would not be able to achieve its objectives. By leveraging strategic communication within the Ministry of Health, the team was able to argue successfully for more staff positions, framing it as an appropriate response to the extent of the malaria problem in the country. Within a few years, the program grew to employ approximately 30 people. This level of staffing has elevated the malaria program’s visibility and increased its capacity to do more work.

### Component #4: The medium

“At one point, the Minister of Health mostly talked about maternal, child health and tuberculosis. And she was so busy it was hard to get a meeting with her, even though there was a raging malaria upsurge. Showing her the map of malaria cases, highlighting the number of deaths, when I encountered her in the hallway one day allowed me to quickly and starkly show her the extent of the problem. Then I was able to secure her support for an urgent response.”

As noted, strategic communications can be formal or informal, verbal or nonverbal. Selecting the appropriate medium for communicating with a given stakeholder—that is, formulating a version of the malaria program’s message that is applicable to their concerns and interests, identifying the kind of information they need to fulfill their functions, and understanding how they prefer to receive information—is essential. For example, in order to do his or her job, a policy analyst may need to receive a briefing that provides an interpretation of data from a study of availability of malaria medications in public health care facilities, but she may not need to review the data as an academic might. A journalist, on the other hand, may prefer to learn about the story of a single person affected by lack of access to malaria medications, a story that could serve as a “hook” for an article. Members of a village health team may need to get a briefing on the study’s main findings conducted in a local language. And a politician rushing from a meeting to a public appearance may only have time to listen to a three-sentence “elevator pitch” and glance at a simple infographic based on the study data. Concisely stating the problem looks different for each audience; pairing the description of the problem with a specific solution gives the malaria program an opportunity to suggest its preferred approach to addressing the challenge.

These examples assume that the malaria program already has access to reach these stakeholders. For those they have not yet made direct contact with, engaging with media outlets, including television, newspapers, radio and social media, may be required to capture their attention. In other cases, it may be more appropriate to identify acquaintances of the target audience in the malaria program’s network and ask them to help with the approach.

### Component #5: Timing and setting

“I decided to attend the conference because I saw that there were several speakers I wanted to connect with. This way, I could find them all and have a quick word, either to get them on board with our new initiative or at least nail down an appointment for later in the month.”

The fourth element of successful communications is understanding when and where to deliver key messages. For example, it is important to understand the country’s budgeting and planning cycles. Approaching policy makers with a great proposal the day after that budget has been approved will not garner the program support. Similarly, in the middle of a national election campaign, newspapers may not want to print a malaria story on the front page.

A related aspect is the setting for delivery of a message. For example, the malaria program manager may take advantage of less formal social venues, like sports and Rotary Clubs, churches, or social functions. In these locales, stakeholders may be in receptive moods, enabling them to better receive key messages.

Sometimes strategic timing requires knowing when to back off and return to a topic later. Approaching a policy maker or industry leader in a public forum to discuss a controversial decision may not be strategic timing; in this case it may be better to request a private appointment instead. The importance of timing means that the malaria program manager must keep in mind which messages need to be communicated to which stakeholders, so that if an opportunity arises unexpectedly, he or she can jump straight to the point.

## Discussion: Building a chorus of voices on malaria governance

“If a woman dies while delivering a baby, there will be an outcry from the community and an investigation into root causes. But if someone dies of malaria, it’s just normal business.”

Making malaria a “hot topic” will not happen overnight, nor will it happen if the national malaria program is the only advocate. Messages about the importance of focusing on malaria cannot come only from health professionals. Politicians and policy makers who only hear complaints about malaria from the staff of the malaria program may infer that it is not a major problem or that program staff are just angling to keep their jobs. Thus the malaria program must reach out to a wide array of stakeholders to include them in the program’s communications activities and empower them to undertake their own activities independently.

One key group in this effort is journalists, whose core mission is updating the public about pressing issues. In Uganda, the malaria program has provided trainings and information sessions for journalists to inform them about the state of malaria in the country. Following these formal engagements, journalists have come to understand the importance of a coordinated effort to address malaria as an urgent priority. They are now eager to answer informal phone calls from malaria program staff and attend their press conferences. Their coverage keeps malaria on the front pages for the public’s attention, and their questions to politicians keep the issue at the forefront of policy debates. Indeed, before the COVID-19 pandemic began, malaria was receiving the most coverage of any disease in Uganda, keeping it in the public eye and emphasizing its seriousness. While COVID-19 has altered global and national priorities around the world, malaria remains a major problem that, if it is not addressed, will experience an upsurge.

Finally, the most important strategic communications must come from the people directly affected by malaria. The malaria program must not only provide communities with services; it has to listen to their lived experiences, help them to identify likely solutions, and then organize to make their messages heard. As one Ugandan malaria advocate said: “When a clinic runs out of anti-malarial medicines, it should create the same uproar among patients and civil society groups as a stock out of HIV antiretrovirals does.” The malaria program cannot and should not do strategic communications in isolation. It has to recruit and engage other communicators—first to help them articulate the impact of malaria on their lives and health, and then to support them to do their own strategic communications about the disease. These stakeholders may include civil society organizations, patient and child rights advocates, labor unions and other industry partners, and local, religious, and traditional authorities. By developing messages and messengers collaboratively, the malaria program and its stakeholders can coordinate to make their voices heard.

## Conclusion

Governance entails setting overarching goals, marshalling resources, and holding actors accountable for working towards achieving intended results. All of these governance activities involve human beings. And all human beings, and the systems we create, have our own interests. Strategic communications helps us to listen to and understand others’ interests so that we can explain to them where our interests intersect and overlap. By doing so, we can more effectively influence policy and decision making. In the case of malaria, national malaria programs need to communicate that malaria is both an urgent problem and that it has feasible solutions. Strategic communications help malaria programs “sell” the message about why and how best to address the complex challenges of malaria. Yet our literature review found no published papers examining strategic communications by national malaria programs. In response to this gap, we have therefore proposed a framework for developing and studying strategic communications for malaria governance in endemic countries.

Strategic communications for governance should be included in all leadership development opportunities for national malaria program managers. It is a natural extension of skills that are often included in leadership training, such as stakeholder analysis, decision making, change management, identifying multipolar dimensions of performance, and understanding the politics of organizations and their environment. Communication skills help bridge the gap between analysis and action by giving malaria program managers the capacity to persuade stakeholders to support the program to achieve its objectives. As mentioned, communications is a “soft skill.” There are no pat formulas for successful communication. Indeed, one feature of successful leadership is noticing what works and developing “gut feelings” about how and when to communicate with different stakeholders.

Malaria program managers need more routine opportunities to share their experiences with successful communications among themselves. Currently, most contact among national malaria program managers is mediated through international agencies and academic institutions. Efforts to support direct, “south-south” communication and learning should be fostered to enable a community of practice among practitioners that does not need to be facilitated or moderated by partners from non-endemic countries. For example, initiatives such as the ALMA Scorecard for Accountability and Action from the African Leaders Malaria Alliance (ALMA) provides senior leadership with up-to-date information on the fight against malaria, fostering transparency and accountability to stakeholders [6]. This and other efforts create opportunities to for strategic communications to improve governance at local, national, and regional level.

Ultimately, strategic communications for malaria governance represent a key approach to restoring global progress on the path to malaria elimination. We offer the framework and discussion presented in this paper as a first step towards strengthening and understanding the strategic communications approach.

## Supporting information

S1 Text "Rethinking Malaria in the Context of COVID–19," a global engagement organized by Harvard University. (DOCX)
