# Supplementary material for: Rethinking communications for governance of malaria programs
Source: PLOS Glob Public Health. 2023 Jul 31;3(7):e0001132. doi: 10.1371/journal.pgph.0001132 (PMC10389711; doi:10.1371/journal.pgph.0001132)
Supplement: S1 Text — (DOCX) [file pgph.0001132.s001.docx]

**S1: "Rethinking Malaria in the Context of COVID–19" was a global engagement organized by Harvard University.** For more information, see:

Rethinking Malaria/COVID19 [Internet]. Boston, MA: Harvard University Defeating Malaria: From the Genes to the Globe Initiative; 2021. [Accessed 13 July 2023] Available from: <https://www.defeatingmalaria.harvard.edu/rethinking-malaria/>.
